# Ultrafast time-resolved X-ray absorption spectroscopy at FemtoMAX beamline

**DOI:** 10.1107/S1600577526006363

**Published:** 2026-07-27

**Authors:** Van-Thai Pham, Katharina Kubicek, Ahn Byungnam, Carl Ekström, David Kroon, Zhangatay Nurekeyev, Mohammed Sekkal, Axl Eriksson, Hyein Hwang, Anna Zymaková, Thanh Binh Nguyen, Minh-Huong Ha-Thi, Xuan Thang Vu, Yohei Uemura, Jörgen Larsson, Christian Bressler, Jens Uhlig

**Affiliations:** a MAX IV Laboratory Lund University PO Box 118 SE-221 00Lund Sweden; b European XFEL GmbH Holzkoppel 4 22869Schenefeld Germany; c Department of Physics Universität Hamburg Luruper Chaussee 149 22761Hamburg Germany; d The Hamburg Centre of Ultrafast Imaging, Luruper Chaussee 147, 22761Hamburg, Germany; e Department of Chemical Physics Lund University 22362Lund Sweden; f ELI Beamlines Facility, The Extreme Light Infrastructure ERIC, Za Radnici 835, 25241Dolni Břežany, Czechia; g Institut de Chimie des Substances Naturelles, CNRS UPR 2301 Université Paris-Sud, Université Paris-Saclay 1 Avenue de la Terrasse 91198Gif-sur-Yvette France; h CNRS, Institut Des Sciences Moléculaires d’Orsay Université Paris-Saclay 91405Orsay France; i Institute of Materials in Electrical Engineering 1 RWTH Aachen University Sommerfeldstr. 24 52074Aachen Germany; j Department of Physics Lund University PO Box 118 SE-221 00Lund Sweden; University College London, United Kingdom

**Keywords:** ultrafast X-ray absorption spectroscopy, iron tris bi­pyridine, femtosecond X-ray pulse, timing tool, XANES, EXAFS

## Abstract

We report on the development and first experiments at a new X-ray absorption spectroscopy (XAS) endstation at the FemtoMAX beamline of the MAX IV Laboratory. Femtosecond time-resolved XAS measurements on solvated photoexcited molecular systems were performed with pulse-width-limited temporal resolution. The results demonstrate nearly shot-noise-limited signal quality, highlighting the capability of this endstation to extract detailed electronic and geometric structural information.

## Abstract 2

A new setup for femtosecond resolution X-ray absorption spectroscopy with energy ranging from 2 to 15 keV is established.

## 1. Introduction

Historically, the continued development of advanced light sources, such as synchrotrons and free-electron lasers, has significantly advanced our understanding of atomic-scale matter over the past 70 years. X-rays, with their ability to penetrate matter and probe specific atoms, have enabled us to gain insight into the structure of complex systems such as molecules and proteins (van Bokhoven & Lamberti, 2016 ▸; Drenth, 1999 ▸). However, a static structure is an averaged coordination of atoms, which move, react and transform constantly. To track these activities, time-resolved techniques have been applied to study molecular dynamics. With the development of ultrafast optical laser systems, time-resolved studies at synchrotrons with a temporal resolution on the order of 100 ps have made it possible to study the later stages of photophysical and photochemical processes (Chen *et al.*, 2014 ▸; Gawelda, Pham *et al.*, 2007 ▸; Haldrup *et al.*, 2012 ▸; Saes, Bressler *et al.*, 2003 ▸; Zhang *et al.*, 2007 ▸; Gawelda *et al.*, 2005 ▸). Soon thereafter, studies showed a temporal resolution on the order of 200–300 fs at synchrotrons enabling a unique view on the earlier dynamics (Bressler *et al.*, 2009 ▸; Schoenlein *et al.*, 2000 ▸; Stamm *et al.*, 2007 ▸). The emergence of the X-ray free-electron laser (XFEL) brought ultrashort X-ray pulses with durations as low as a few tens of femtoseconds at a significantly higher flux. This made it easier to explore the dynamics of processes, including charge transfer processes involving the local solvation shell (Vester *et al.*, 2022 ▸), molecular vibrations (Lemke *et al.*, 2017 ▸; Katayama *et al.*, 2019 ▸), or even the study of natural systems that perform photosynthesis (Huijser *et al.*, 2018 ▸; Kunnus *et al.*, 2020 ▸). These studies in turn aided the design and development of even more advanced materials, *e.g.* towards highly efficient photovoltaic devices and photocatalysis based on first-row transition metal compounds (Ponseca *et al.*, 2017 ▸).

Among the available ultrafast X-ray techniques, optical-pump/X-ray-probe methods using X-ray diffraction (XRD) and X-ray absorption spectroscopy (XAS) probes were among the first to be used (Bressler & Chergui, 2004 ▸; Britz *et al.*, 2020 ▸; Van Driel *et al.*, 2015 ▸). XAS measures X-ray absorption of a unique atom type in the system without requiring a long-range structure in the system, making it a powerful tool to study disordered systems like molecules dissolved in liquids. Time-resolved XAS measures the transition probability from a well known (core) electronic state into unoccupied electronic orbitals and is thus particularly sensitive to changes in oxidation state during a redox reaction (Gawelda *et al.*, 2006 ▸); Bressler & Chergui, 2004 ▸).

The ultrafast temporal resolution can help resolve ultrafast light-induced processes in real time (Bressler & Chergui, 2004 ▸). In transition-metal complexes in solution, such as the iron complex studied in this paper, XAS measures the electron density at the iron metal center and thus directly measures the charge transfer from the metal to the ligand. The primary alternative technique would be X-ray-induced *K*α or *K*β emission spectroscopy, in which the signal arises indirectly from the spin–orbit coupling of the transferred electrons, with the spin, distance and coupling parameter contributing to the signal.

FemtoMAX bridges the gap between the low-flux femtosecond slicing sources at synchrotrons and the intense X-ray free-electron lasers: it uses the 100 fs electron bunches produced with 10 Hz from the linear accelerator (LINAC) of MAX IV. A photo gun generates bunches, which are then compressed and sent through two undulators to produce spontaneous X-radiation, which is guided into the FemtoMAX beamline located below the main storage ring. The generated X-rays maintain the 100 fs pulse duration of the electron bunch (Enquist *et al.*, 2018 ▸). Its single-pulse intensity is typical for synchrotron radiation undulators. It lies in the range 10^6^–10^7^ photons pulse^−1^ (scaled to a 1% energy bandwidth), which is 10^6^–10^7^ times lower than XFELs, as the spectrum simultaneously covers the tender and hard X-ray ranges (1.8–15 keV).

The pulse-to-pulse intensity fluctuations vary by 10% peak-to-peak, with a typical RMS value of 2%, which relaxes the demand on monitoring incoming X-ray intensity. This source thus combines short pulses, stable flux and an extensive energy range, at a repetition rate where sample movements that refresh the sample between pulses are easy to accomplish.

We have developed an XAS endstation for laser-pump/X-ray-probe experiments at FemtoMAX beamline. In a benchmark experiment with [Fe^II^(bpy)_3_]^2+^, we used total-fluorescence-yield (TFY) detection mode. Measurements at XFEL sources typically employ Si(111) monochromators. We employed InSb(111) with its two to three times larger bandwidth, thus trading spectral resolution for higher flux. We show that the constraints on the (current) FemtoMAX flux can be mitigated by photon-statistics-limited detection and by maximizing the fluorescence collection efficiency. We show that it approaches shot-noise-limited signal quality and allows the extraction of structural dynamics information from a photoexcited molecule.

## 2. Experimental setup

### 2.1. The X-ray spectroscopic endstation

X-rays generated at the FemtoMAX beamline are focused by a toroidal mirror onto different longitudinally placed endstations. The new spectroscopy endstation was located 8 m downstream from the toroidal mirror, and at the time of the experiment the FWHM X-ray spot size was 150 µm × 170 µm (horizontal × vertical) at the sample position. The spot size has since been significantly reduced (FemtoMAX beamline webpage: https://www.maxiv.lu.se/beamlines-accelerators/beamlines/femtomax/).

The endstation was designed to be compact and fit between the other endstations. It includes a dedicated chamber that is capable of holding a vacuum, but was operated in an atmospheric helium environment to accommodate the samples. The chamber can move vertically and horizontally, orthogonally to the beam direction, using motorized stages. The entire setup is mounted on a 60 cm × 40 cm breadboard, which holds the translation stages and optical components. The sample environment is connected to the upstream X-ray beamline vacuum through a thin Kapton foil vacuum window. The atmospheric helium environment allows for easy operation of a liquid jet even in the tender 2–5 keV energy range. The entire sample chamber, including its tube connections, is gently flushed with helium via a liquid-seal bubbler, which maintains a solvent-saturated helium atmosphere.

**Figure 1:**
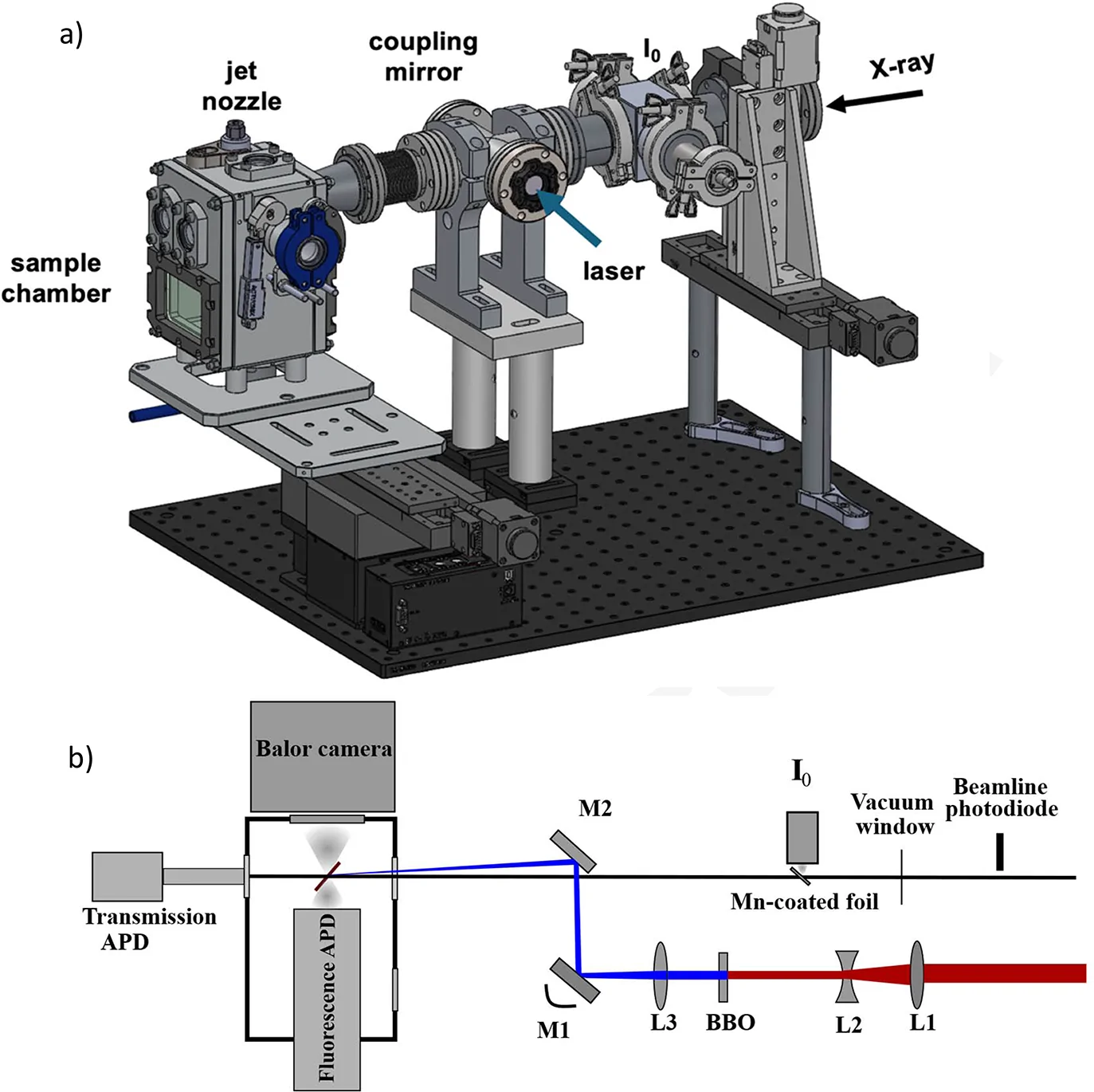
(*a*) A simplified CAD model of the spectroscopy endstation. (*b*) Top view of the endstation. L1 (*f* = 200 mm) and L2 (*f* = −100 mm), which reduce the initial laser beam size, and L3 (*f* = 700 mm) are focusing lenses, M1 and M2 are dielectric mirrors at 400 nm, where M1 is motorized and M2 is inside a four-way cross mirror housing. A vacuum window separates the helium environment with the beamline vacuum which accommodates a photodiode to measure the X-ray flux, and *I*_0_ measures the relative incoming X-ray intensity for every X-ray pulse during the pump–probe measurement.

We measure the relative incident monochromatic X-ray flux (*I*_0_) for energies above 6 keV right after the Kapton window through fluorescence from a thin Mn-coated Kapton foil mounted at 45° to the incoming beam (Fig. 1 ▸). Its 0.8 µm-thick Mn layer was tailored to absorb ∼20% of the incident flux at 7.1 keV. An avalanche photodiode (APD, from Advanced Photonix Inc.) collects the weak Mn *K*α fluorescence. Due to the high bias voltages, we isolated the APD from the He atmosphere to avoid electrical arcing. This allows us to safely operate the APD at its elevated bias voltage of 1.7 kV.

The flat sheet liquid jet used in this experiment was produced by a rotatable sapphire nozzle (Kyburz, Switzerland). The rectangular-shaped sapphire orifice creates a homogeneous, 100 µm-thick, flat sheet with a width of approximately 8 mm at a flow speed of approximately 6 m s^−1^. The flat sheet plane is rotated 45° relative to the incident X-ray beam to enable both efficient collection of X-ray fluorescence and measurement of the transmitted X-ray beam with APDs. The sample was continuously cycled using a small gear pump. In the current configuration, the minimum sample volume can be as low as ∼30 ml, limited by the volume of the reservoir used. A video camera continuously monitors the flow of the liquid jet and the state of the reservoir.

The sample position inside the chamber is fixed, and the entire chamber is moved relative to the laser and X-ray beams.

An X-ray fluorescence APD (C30703FH, Excelitas Canada Inc., 1 cm × 1 cm active area) is positioned approximately 1 to 2 cm from the center of the jet sideways at 90° to the incoming X-ray beam to record the TFY. To maximize TFY collection, the utilized jet nozzle is designed such that the lower part of the sapphire core (close to the jet opening) is not enclosed by the bulky (1-inch diameter) steel housing (and also containing Fe) to allow a very close approach of the APD to the interaction region, thus increasing the solid angle for collection. In the current setup, the closest sample-to-detector distance is merely limited by the need to suppress scattered pump-laser light.

A larger secondary window [Fig. 1 ▸(*b*)] allows the placement of either a direct-detection partial-fluorescence detector or a crystal spectrometer. It was initially designed to place the sCMOS (Balor camera) X-ray detector from the beamline with the detector housing as close as 35 mm from the interaction point and thus its sensor chip as close as 45 mm from the source of fluorescence, which corresponds to a solid angle of 4.7% (compared with 8% of the TFY). Direct energy detection on a large-area detector would enable single-photon counting and separate fluorescence from elastically scattered X-rays and optical pump laser leakage. During these commissioning experiments, the camera was not available for operation in this mode. The same exit port could also be used to detect and analyze the X-ray fluorescence with either a crystalline energy analyzer (Alonso-Mori *et al.*, 2015 ▸) or a microcalorimetric energy-sensitive detection scheme with much higher energy resolution (Uhlig *et al.*, 2013 ▸).

For transmission experiments, a second APD is connected to the back of the chamber via a 30 cm extension tube. The extension tube increases the distance to the sample, thereby increasing the spatial separation between the laser pump and the X-ray probe. Only the transmitted X-ray beam exits the extension through a Mylar-sealed hole in a vacuum-blind flange, significantly reducing recorded light scatter from the laser and protecting the light sensitive APD.

To shield the two APDs from the pump-laser radiation, two foils were mounted on their entrance windows. One is an 8 µm-thick Al foil, and the second is a Mylar film, coated with 0.4 µm of Al to cover any pinholes in the thicker film. While providing strong optical shielding, the foils attenuate the X-ray flux in the 6–8 keV range by only ∼17%. They can be adjusted to the photon energies used in the specific experiment.

The XAS chamber and the optical laser beam paths nearby were designed with a wide range of experiments in mind. Additional windows for the chamber, for example, allow the collection of wide-angle scattering patterns with an opening angle of up to 80° or the adjustment of the excitation angle between the pump and probe beam to 54°. In the collinear geometry used in this experiment, the temporal resolution of a system is limited by the group velocity difference between the optical pump and the X-radiation probe in the sample. This effect, approximately 1 fs per 1 µm of sample thickness, can significantly impact the achievable instrument response function (IRF) for thick samples.

At 42°, the group velocity difference between the optical pump and X-ray probe through the liquid jet is compensated. In this geometry the projection difference over the projected spot size is defining the temporal resolution. The recent paper by Carbone and co-workers suggests an approach using wavefront tilting to remove the broadening introduced by a larger projection angle (Pennacchio *et al.*, 2017 ▸). This approach would theoretically permit the use of thicker jets at lower concentrations, which is in some cases desirable where, for example, the accessible amount of precious sample molecules is limited. Without wavefront tilting the work at 42° with a flat wavefront would introduce an approximate 185 fs delay for a 80 µm spot size independent of the jet thickness. While wavefront tilting of the optical pump beam is challenging, recent advances in, for example, the THz field have yielded deployable designs (Tóth *et al.*, 2023 ▸)

### 2.2. The optical laser system

The optical laser is located on a floor above the beamline, and its 800 nm beam is guided through an evacuated transfer system down into the X-ray hutch. In this experiment, we used a 10 mm-diameter BBO crystal with a 0.2 mm thickness to frequency-double to 400 nm and filtered the residual 800 nm light using two dichroic mirrors. A 700 mm focal length lens focuses the beam behind the sample and creates a ∼180 µm × 240 µm (horizontal × vertical) FWHM spot size on the sample, with an adjustable pulse energy up to 0.25 mJ. A motorized mirror [M1 in Fig. 1 ▸(*b*)] permits the steering of the beam that excites the sample at an angle of approximately 3°. The last mirror [M2 in Fig. 1 ▸(*b*)] is located inside a cross that is decoupled from the chamber via a bellow. The distance from the mirror to the sample is approximately 40 cm. Details are given in Fig. 1 ▸.

## 3. Spatial and temporal overlap

### 3.1. Spatial overlap

The sample is provided by a flat-sheet sapphire nozzle mounted inside a 1-inch stainless steel cylindrical housing atop the sample chamber. The initial spatial overlap between the pump and probe beams on the sample is achieved by mounting a windowless CCD camera in the same mount, with the sensor plane centered on the rotation axis and at the same position as the flat jet center. The windowless operation of the camera enables detection of both the laser and X-ray beams, and its 3.45 µm pixel size enables precise beam alignment and spot-size measurement for each beam, which are typically 100–400 µm in diameter. This method permits spatial overlap of both beams with immediate feedback. However, it requires the introduction of several neutral-density filters into the laser beam path, the prior removal of the sample system, and opening the chamber to ambient air. The chamber is equipped with an exchangeable <1000 µm pinhole that can be introduced into the beam without opening the chamber or removing the sample. In these initial experiments, we faced challenges with the laser mode and thus relied on more infrequent checks with the CCD camera, which allows for more nuanced alignment. We estimate an alignment precision of ±20 µm.

### 3.2. Temporal overlap

**Figure 2:**
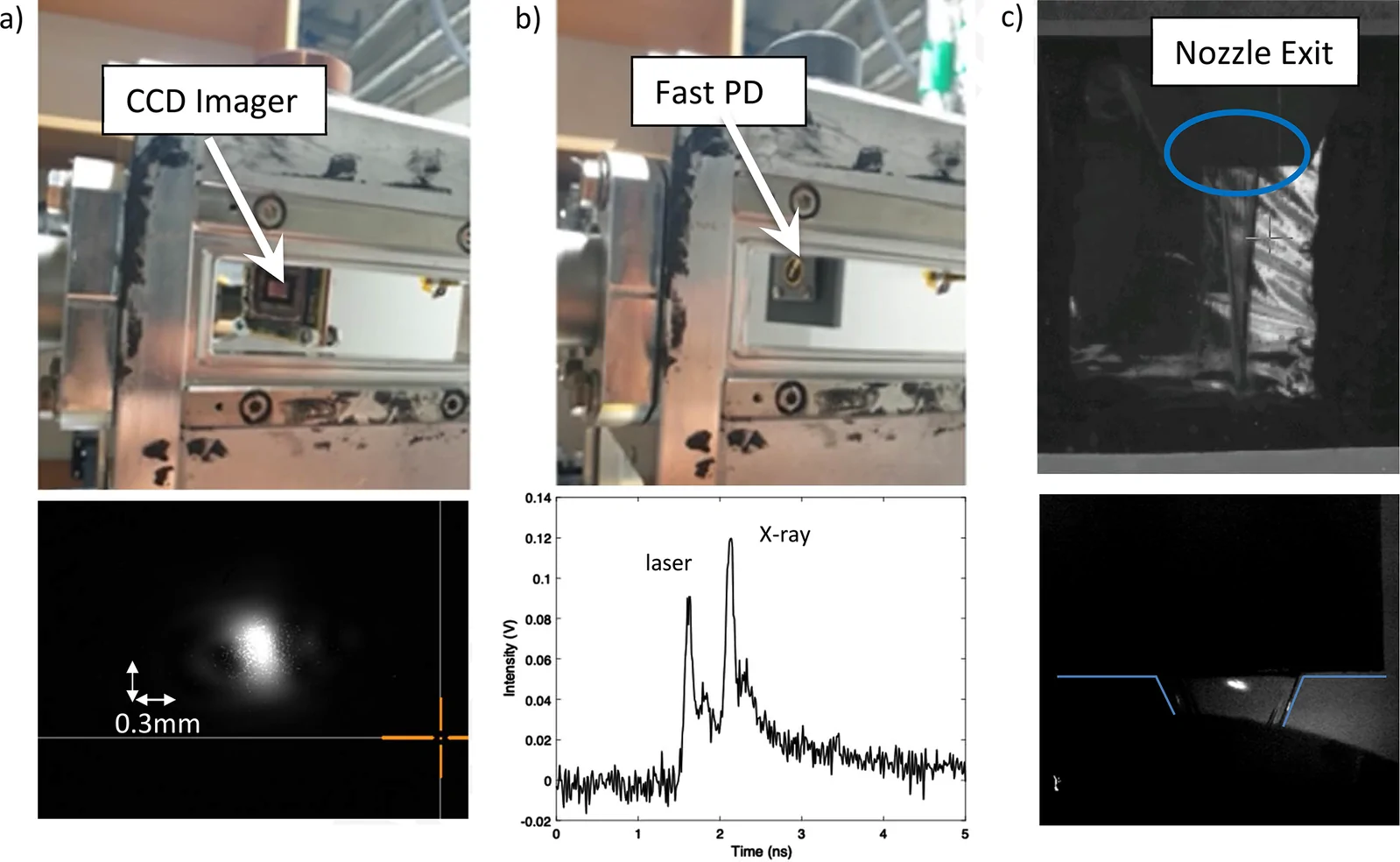
(*a*) The upper photograph illustrates a windowless camera mounted exactly at the sample position. The lower image shows overlaps of laser and X-rays monitored by the camera. (*b*) The upper photograph illustrates a windowless fast photodiode mounted exactly at the sample position. The lower figure shows a two-pulse signal that represents the laser and X-ray pulses measured by the fast photodiode. (*c*) Position of the mounted liquid flat-sheet jet at the same position as the previously mounted camera and diode. The top image shows a larger view; the bottom shows a zoomed-in view of the blue oval line with the excitation laser on the jet. The blue line is a guide for the eye showing the border of the liquid and the nozzle housing.

For coarse temporal overlaps, we use a fast Hamamatsu G4176-03 diode with a 30 ps rise time that measures the laser and X-ray pulses simultaneously. The diode is mounted on a 1-inch holder at the sample jet nozzle position [Fig. 2 ▸(*b*)]. Its signal is extracted over a bias T, which also supplies the required 10 V bias voltage to the diode. A 36 GHz oscilloscope samples the extracted analog signal. By tuning the laser phase shifter, the temporal overlap between the laser and the X-rays can be directly observed on the oscilloscope waveform with approximately 100 ps precision. Through interpolated fine-stepping of the delay, an accuracy of 1 ps is achievable. Relative shifts in timing are measurable from this point on using the timing tools discussed in the next section.

### 3.3. Timing tools and temporal resolution

FemtoMAX uses two independent timing tools to measure the arrival time of each X-ray pulse relative to that of each laser pulse. The RF-cavity-based jitter monitor tool measures electron arrival time with respect to the laser, achieving 200–300 fs accuracy and an extensive scan range. The second timing tool uses sum-frequency generation (SFG) between white light produced from the electron beam dump magnet at the end of the LINAC and the laser, in a cross-correlator setup with 70 fs (FWHM) accuracy. The details of the RF and cross-correlator timing tool are reported by Enquist *et al.* (2018 ▸) and Kroon *et al.* (2025 ▸), respectively. During these commissioning experiments, both timing tools were available.

The RF timing data are acquired from the fast 36 GHz oscilloscope while data from APDs are acquired using a slower oscilloscope. The cross-correlator data in the form of images are acquired from an intensified CMOS camera. All data are synchronized using a common gated trigger signal, ensuring that they correspond to the same X-ray pulse.

The RF timing data are recorded in a digital format and can be directly used as a relative time stamp between the laser and X-ray pulses. In contrast, the cross-correlator signal is obtained as an image of the SFG beam, which is analyzed to extract the laser arrival time in pixel units; this is then calibrated using the RF timing data to obtain a high-precision time stamp.

The temporal resolution is dominated by group-velocity mismatch (GVM) between the optical pump and X-ray probe in the 100 µm liquid jet tilted by 45°, resulting in an effective instrument response of ∼145 fs in the collinear geometry. A non-collinear geometry (pump–probe ≃ 50°) is implemented in the endstation design. This geometry compensates GVM by matching optical and X-ray path lengths through the jet, reducing the effective IRF to below 50 fs for a 100 µm jet. However, at the time of the experiment this was not implemented but rather a target for future improvement.

The timing jitter during the measurements was below 1 ps, with occasional drifts of a few picoseconds over longer acquisition periods. The data were sorted into 50 fs bins, which are smaller than the intrinsic timing uncertainty and therefore mainly serve as a fine sorting interval. The energy scan range from the monochromator (∼60 eV) introduces only 24 fs of additional delay at the maximum energy of the scanning range. At the time of the experiment, no active timing stabilization or feedback was implemented but it will be a point of improvement in the future.

## 4. Data acquisition and signal extraction

During the experiment, we simultaneously measured the transmitted and absorbed X-ray intensities via X-ray-induced fluorescence. The fluorescence APD is amplified by a trans­impedance amplifier with a gain of 10^4^. This amplified signal is then digitized by a fast oscilloscope (4 GHz analog bandwidth, 8-bit dynamic range, Lecroy Waverunner) and recorded as a time trace by the MAX IV DAQ control system. Ground loops and electronic pickup noise in the analog signal are reduced by carefully grounding the amplifier, avoiding ground loops, and using short, doubly shielded BNC cables to connect the amplifier to the oscilloscope. The diodes’ responses to each photon bunch show a peak, followed by some ringing. We tested different methods to evaluate the signal from the recorded waveform. Interpolating the waveforms with shapes, such as polynomial functions, or using optimal filters created by averaging the pulse shape and scaling it to the measured shape, increases the correlation between the flux measured by the different detectors slightly over simple integration of the central peak height. These approaches take the shape of the response functions into account and, as such, represent the detector’s physical response better. However, while these improvements are observable, they were not the noise-limiting factor in our experiments, and we opted to use the box-car-integrated central peak area as a measure of the photon flux.

A standard acquisition scheme for TR-XAS is used, in which the laser excites the sample with every second X-ray pulse (Saes *et al.*, 2004 ▸; Lima *et al.*, 2011 ▸). The X-ray fluorescence from the unpumped sample (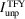) and laser-pumped sample (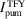) are normalized by their respective incoming X-ray intensity *I*_0_. Each pulse has a stamp noting the selected incoming energy (monochromator setting), the relative arrival time derived from the set time and timing tool measured time-shift, and a flag that indicates whether the X-ray pulse was probing the laser-excited or unexcited state, and the data are sorted accordingly.

We performed two types of scans. One during which the energy was varied at a set time point, and a scan during which the energy was kept at a fixed monochromator position and the relative timing between laser and X-rays was varied.

During scans in which the relative arrival time was varied, each measured pulse pair was time-sorted into 50 fs time bins and then averaged.

During scans in which the energy is varied at a fixed time delay, only X-ray pulse pairs with timestamps within ±1.5 ps of the set value are accepted. The resultant transient signal is then energy-sorted and binned into one 1 eV-wide bins.

The normalized difference signal between the pumped intensity 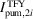 and the unpumped intensity 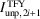 of the *i*th pulse can thus be expressed as
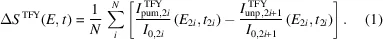
General data acquisition is controlled by a PandaBox (Zhang *et al.*, 2017 ▸), which is triggered by a 10 Hz signal from the LINAC. The PandaBox generates multiple Transistor–Transistor Logic (TTL) output signals that are written into the data and used to signal, for example, a ‘laser on’ or ‘laser off’ state. During these experiments, we operated at half the repetition rate of the input X-ray trigger signal and used a pulse picker that governs the pumping laser’s repetition rate. The pulse picker would allow other, more advanced, pumping schemes.

## 5. Beamline flux and signal quality

We measured the signal quality of TR-XAS measurements as a function of incident beam intensity using the statistical method described by Saes *et al.* (Saes, Gawelda *et al.*, 2003 ▸). The goal of the method is to assess whether the acquired signals are primarily limited by Poisson statistics based on the number of detected photons (shot noise) or whether additional noise sources degrade signal quality. We thus compared the measured distribution of individual signal values with the Gaussian distribution expected from the photon number measured upstream by the beamline diode [as shown in Fig. 1 ▸(*b*)].

**Figure 3:**
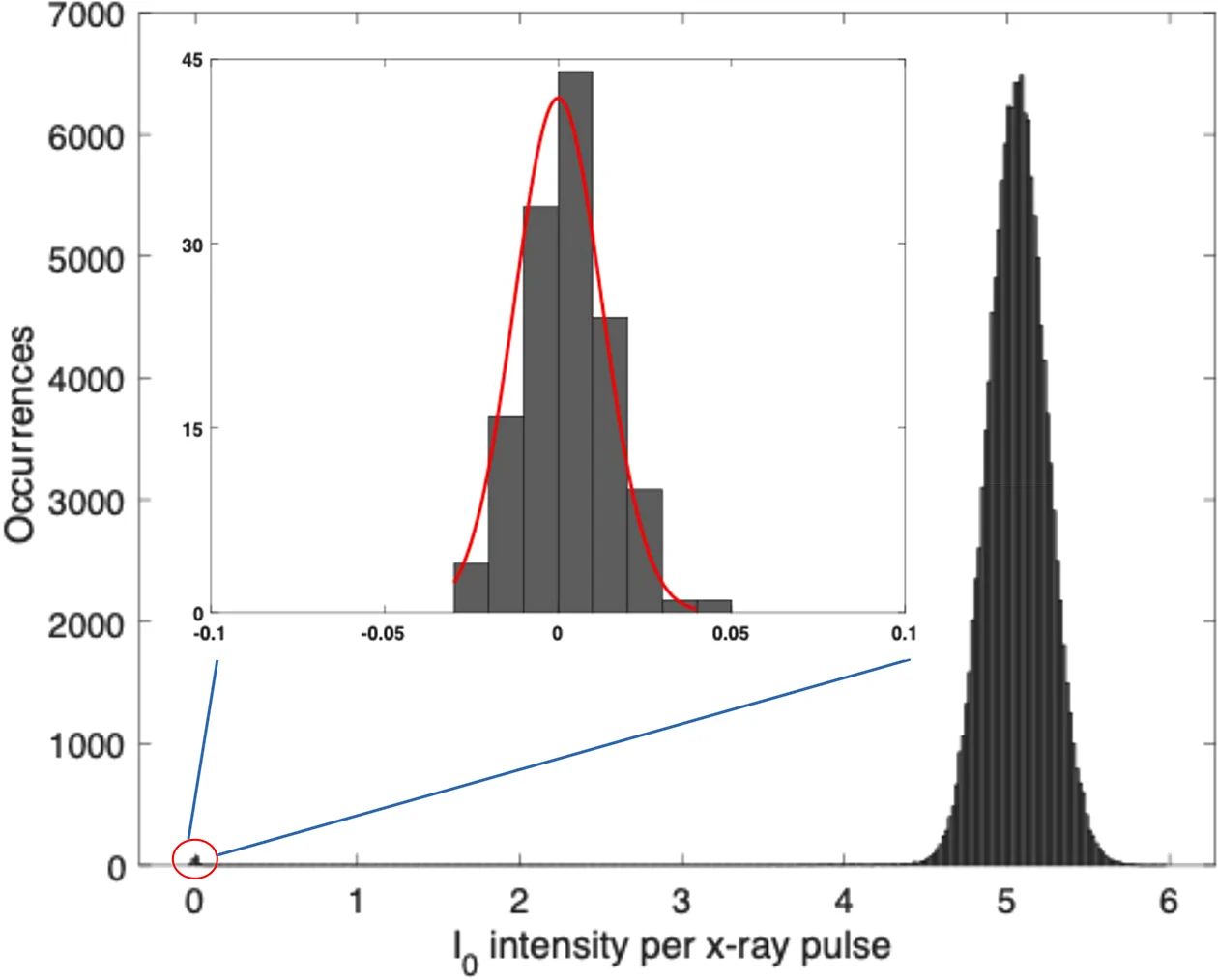
Distribution of the signal of incoming X-ray photons at the *I*_0_ detector, including the dark noise (without X-rays). The inset shows a zoomed-in view of the dark noise distribution (red circle). Here, the noise distribution without X-rays is 14 times smaller than with X-rays.

We start by examining the intensity distribution of recorded X-ray pulse signals on the *I*_0_ APD detector (Fig. 3 ▸). Its Gaussian-shaped distribution contains shot noise σ_p_ (Poisson statistics) and all electronic noise contributions σ_e_ via
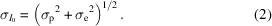
The dark noise is of purely electronic origin (inset of Fig. 3 ▸) and has a distribution approximately 14 times narrower than that of typical X-ray signals. We calculate the effective number of photons per pulse (as delivered by the measured X-ray pulse signal distribution) via
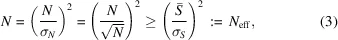
where *N* and σ_*N*_ represent the average number of photons per pulse incident on the detector and its standard deviation, respectively, 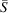 and σ_*S*_ denote the average measured signal and its standard deviation, respectively (Saes, Gawelda *et al.*, 2003 ▸). The signal here corresponds to the incident intensity *I*_0_ and 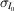. Equation (3) contains an additional noise source for the measured signal; therefore, *N*_eff_ is always smaller than or (at best) equal to the actual number of photons *N*. With this method, we found an effective number of incident photons on the *I*_0_ detector of 790 photons pulse^−1^, corresponding to the intensity distribution of the incident beam.

This number can be compared with the photons per pulse calculated from the incident flux from the Mn-coated foil *N*_eff_(TFY) using the detection geometry and fluorescence efficiency to yield a value for the incoming X-ray intensity via
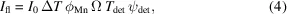
with *I*_fl_ being the fluorescence flux corresponding to *N*_eff_(TFY), *I*_0_ the incident photons per pulse, 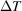 the fraction of incoming flux absorbed by Mn in the Mn-coated foil (0.8 µm), ϕ_Mn_ the fluorescence yield of Mn *K*-emission, Ω the solid angle of the detector, *T*_det_ the transmission through the light-shielding Al foil(s) in front of the detector, and ψ_det_ the detection quantum efficiency of the detector.

The measured transmission of the Mn-coated foil is 80% at 7.1 keV, close to what we would expect for a 0.8 µm-thick Mn foil, where the absorption in the 9 µm-thick Kapton substrate is negligible. This corresponds to a 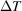 of 0.2. The solid angle for detection on the 16 mm-diameter sensor at a distance of 3 mm from the emission source is ∼0.38. The other parameters for this evaluation are ϕ_Mn_ = 0.3 (Thompson *et al.*, 2001 ▸), *T*_det_ = 0.95, ψ_det_ = 1. This yields an incoming *I*_0_ flux of 3.6 × 10^4^ photons pulse^−1^. This estimation ignores elastic scattering from the foil, which, through horizontal polarization of the X-ray beam and the 90° geometry, is estimated to be significantly weaker. The re-absorption of the fluorescence radiation by the Mn atoms can also be neglected in this estimation.

The photocurrent from the beamline photodiode, which monitors X-ray flux upstream from the Kapton vacuum window of the sample chamber, corresponds to an X-ray flux of 1 × 10^5^ photons pulse^−1^ at the sample position. Some losses and the uncertainty due to the low actual count rates explain the difference, and we can thus assume that the intensity of this order is correct at the *I*_0_ position. We also must conclude that there is still room for improvement, since *N*_eff_ is much smaller.

Based on the measured flux at 7.1 keV, the InSb(111) double-crystal monochromator (DCM) throughput (58%) and the undulator radiation spectrum, centered at 7.1 keV, we estimated a pink beam flux of 3 × 10^7^ photons pulse^−1^.

**Figure 4:**
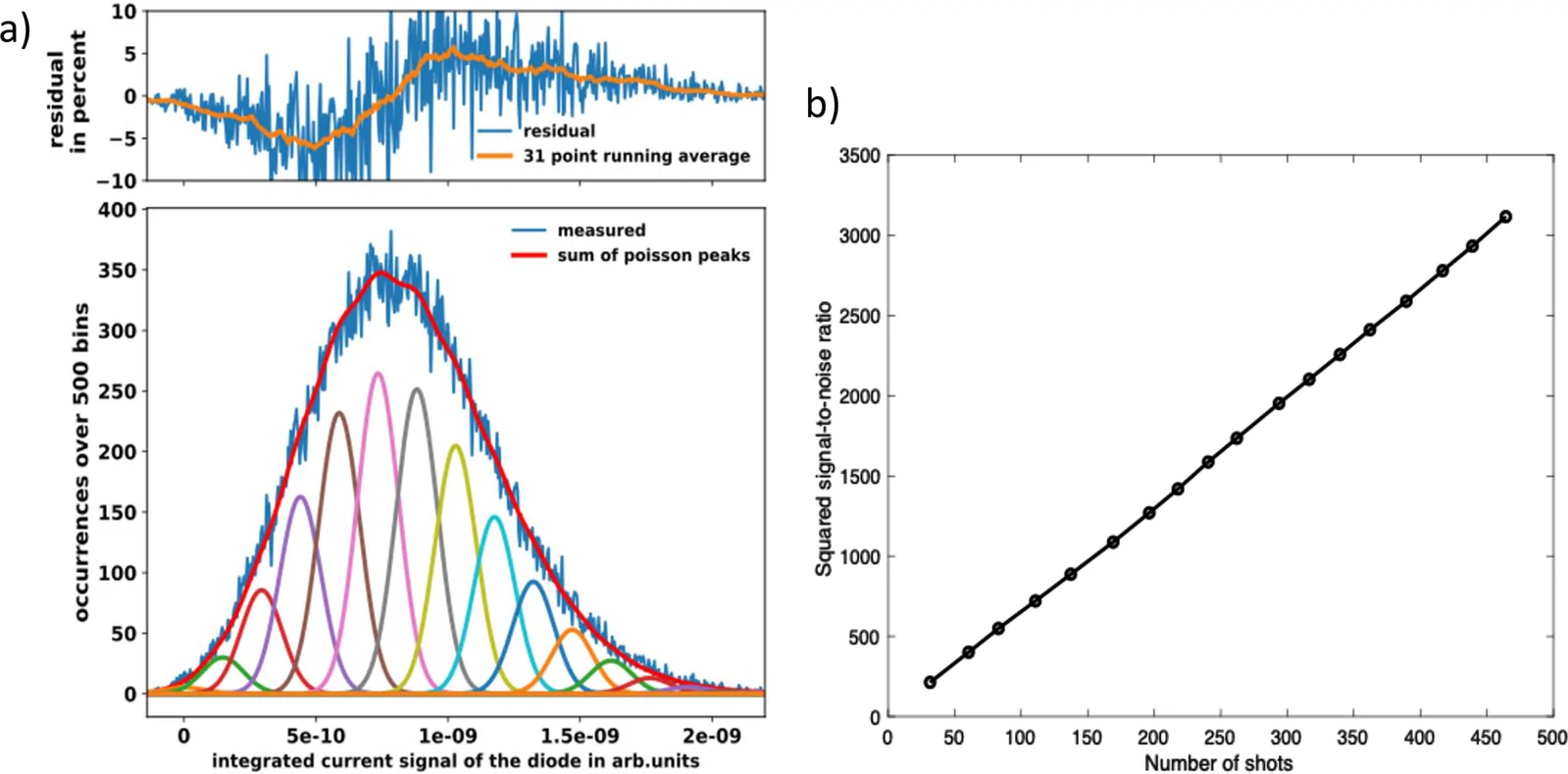
(*a*) Measured intensity distribution of the measured current at the fluorescence APD for 72000 unpumped shots. The lower panel shows a histogram overlaid with a series of Gaussian peaks, each with the same width and an amplitude determined by the probability of a Poisson distribution with a maximum of 5.7 photons. The upper panel shows the residuals of the fit with respect to the measurement. (*b*) Measured/estimated squared signal-to-noise ratio of the fluorescence difference signal versus the number of shots. These data were extracted from a time scan at the B feature where only data from after time zero to 2 ps are analyzed. The slope indicates 6.8 photons pulse^−1^, which is well matched to the flux estimate of the increased fluorescence signal after time zero (see text).

Using equation (4) to estimate the expected TFY intensity distribution from the actual sample yields an average flux of 5.7 photons pulse^−1^ at the B-feature, the dominant transient absorption feature at ∼7122 eV that corresponds to the MLCT (metal-to-ligand charge transfer) → HS (high-spin) transition in [Fe^II^(bpy)_3_]^2+^, of the 50 m*M* sample in water. Using the same approach, we first analyze the stochastic distribution of the measured fluorescence. Fig. 4 ▸(*a*) displays a Gaussian decomposition of the statistical distribution of the recorded fluorescence signal, assuming a Poisson distribution. The relative area of each Gaussian function represents the probability of observing 1, 2, 3, 4, *etc*. photons in the pulse, assuming a maximum of 5.7 photons per shot and a Poisson distribution. The refined (fitted) width for all other events, 2σ, is 1.1 (photons), implying that the FWHM width for each peak is larger than the separation of the peaks. Thus, we cannot distinguish the individual photon events (1, 2, 3, *etc*.) in this histogram, but we clearly see a fine structure that follows the suggested envelope. The other two scaling parameters used in the fit are common to all Gaussians and adjust the summed amplitude and the *x*-axis to the recorded intensity.

We analyzed how the noise of the signal scaled with the number of accumulated shots. The stepwise accumulated signal and the derived signal-to-noise (S/N) are shown in Fig. 4 ▸(*b*) as a function of X-ray pulses. The graph shows that the squared S/N clearly scales with the number of shots, indicating that the number of photons is the main limit on S/N. This underlines the validity of this approach for a single shot, since accumulated pulses clearly move the Poisson distribution into the Gaussian regime.

For the pumped pulses, we noted that a small fraction of excitation laser intensity could leak into the TFY APD (signal during pulses with no X-ray flux). The size of the leak corresponded to one X-ray photon at 7.1 keV corresponding to approximately 2000 electron–hole pairs produced in the APD, which fluctuates with the same distribution as the laser pump power. However, this added noise is small in comparison with the statistical noise.

We note that Figs. 4 ▸(*a*) and 4(*b*) are not from the same scan. Fig. 4 ▸(*b*) shows the statistical analysis (presented as squared S/N ratios) of the ‘laser on’ fluorescence signal in the 200 fs to 2 ps time window as a function of number of shots accumulated. The statistics for the time trace of the ‘laser on’ fluorescence signal indicate a flux of 6.2 photons pulse^−1^ before time zero and 6.7 photons pulse^−1^ after time zero. The ‘laser off’ fluorescence signal shows a statistical flux of 5.3 photons pulse^−1^, most likely due to electronic noise contributions. There is a difference of 0.4 photons pulse^−1^ (5.7 versus 5.3) between Figs. 4 ▸(*a*) and 4(*b*). This could be due to a different solid angle between both measurements. A laser leakage corresponding to 6.2–5.3 = 0.9 photons pulse^−1^ is also calculated from this measurement. The statistical flux of 6.7 photons pulse^−1^ is very close to the 6.8 photons pulse^−1^ value we estimate from the slope in Fig. 4 ▸(*b*).

All of these results are in excellent agreement and show that only minor improvements are needed to reach purely shot-noise-limited photon detection. Modeling the pulse shape around the maximum using a polynomial function, or modeling the whole shape using an ‘optimal filter’, yielded approximately 10% and 15% lower noise (as judged by the stochastic width of the distribution), respectively, but was not yet sufficient.

Table 1 ▸ compares the estimated X-ray flux at the sample position for different detectors and methods used to estimate it. The table reports both the measured flux (directly recorded by detectors) and the calculated flux at the sample position, obtained using equation (4) and accounting for X-ray transmission through either the Kapton foil (for *I*_0_) or the sample.

Among the estimates, the flux derived from the beamline diode is considered the most reliable, as its gain is well known (determined solely by the transimpedance amplifier) and it is based on the integrated current. In contrast, flux values obtained from photon statistics tend to be lower than the integrated electron flux, because additional noise sources broaden the measured distribution, leading to a lower effective count rate. An exception is the *I*_0_ value estimated from sample fluorescence, which yields comparable results for both methods. This agreement can be explained using equations (2) and (3): when the measured flux is very small, noise sources other than photon statistics become negligible, and the integrated electron flux approaches the photon statistics derived value.

To assess the performance of the setup, the incoming photon flux was also estimated from the measured sample fluorescence (Table 1 ▸) using known absorption cross-sections and detection parameters (Table 2 ▸). The flux values reported in the last column of Table 1 ▸ are thus derived quantities that can be directly compared with independently measured fluxes (*I*_0_ from the photodiode). We find that the flux obtained from the fluorescence-based estimate is approximately a factor of five lower than the measured value. This discrepancy is primarily attributed to uncertainties in the effective detection solid angle. During operation, the detector-to-sample distance varied between ∼1 and 1.5 cm to suppress scattered pump-laser light, resulting in a variation of the solid angle by up to a factor of ∼2 (thus from ∼8 to 4%). Additional factors contributing to this difference include partial shadowing of the detector by sample droplets and losses due to residual air absorption when the helium environment is not fully saturated.

The strong deviation of noise statistics coming from the *I*_1_ APD is another clear indication that the noise in this detector is one of the limiting factors. The total area and noise performance of the total fluorescence detection area is indeed one of the factors that could result in the largest improvement. Adding the existing scientific camera from the beamline in single photon detection mode would double the total solid angle without any further modifications to the design. The size and placement of the nozzle is significantly reducing the accessible solid angle. The maximum reasonable accessible solid angle could be achieved with a design using either a rectangular CCD chip or multiple of the currently used 1 cm × 1 cm detectors.

Table 2 ▸ lists the parameters used in equation (4) to obtain the *I*_0_ flux values shown in Table 1 ▸. As can be seen from Table 1 ▸, there is flux missing in the fluorescence and *I*_0_ detector as the derived incident flux is lower than the measured one. We summarize the main contributions to such flux loss and signal limitations as follows:

(i) Statistical/effective flux limitations:

 (*a*) Shot-to-shot fluctuations of the incident X-ray source intensity.

 (*b*) Pump-laser fluctuations affecting excitation stability.

 (*c*) Electronic noise in the detector and readout chain.

(ii) Integrated flux loss and detection efficiency:

 (*a*) Partial air absorption, when helium does not fully fill the transfer line and sample chamber.

 (*b*) Additional X-ray attenuation from optical light-shielding foils.

 (*c*) Limited detector solid angle due to geometrical constraints.

 (*d*) Increased sample-to-detector distance to suppress scattered pump-laser light.

(iii) Additional experimental losses:

 (*a*) Partial shadowing of the fluorescence detector by sample droplets on the detector shielding during long-term jet operation.

Taken together, these factors define the current performance and indicate clear pathways for improvement, which altogether can increase the setup sensitivity by at least an order of magnitude.

**Figure 5:**
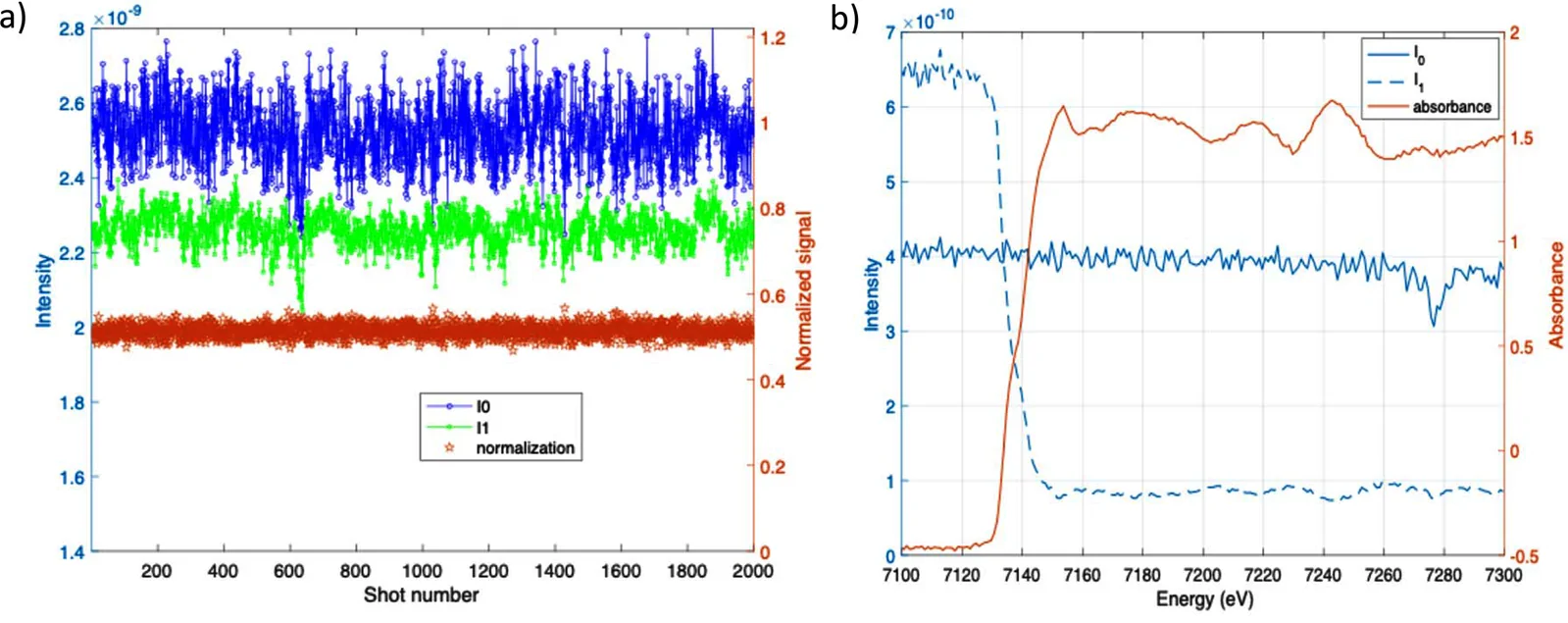
(*a*) Correlation between *I*_0_ and *I*_1_. The left *y*-axis shows the intensity of *I*_0_ (blue) and *I*_1_(green) over time; the right *y*-axis (red) shows the ratio of *I*_1_ and *I*_0_. (*b* Importance of *I*_0_ in the extraction of the absorption spectrum of Fe foil. The left *y*-axis shows the intensity of *I*_0_ (solid line) and that of *I*_1_ (dashed line); the right *y*-axis shows the absorbance of Fe foil which is calculated by abs = ln(*I*_0_/*I*_1_). We note that each Fe foil absorption spectrum is contributed to by 50 X-ray pulses.

The *I*_0_ detector, which measures the incoming X-ray intensity, is essential in all XAS experiments where the incoming flux varies significantly. In most cases, spectral features can only be resolved by normalizing the transmitted signal to the incident intensity. In this experiment, we used an Mn-coated foil to monitor the incident X-ray beam via Mn *K*-shell fluorescence. The detector absorbed approximately 20% of the beam, and the effective count rate was 790 photons per shot. The resulting *I*_0_ signal showed a clear correlation with the transmission signal [Fig. 5 ▸(*a*)]. However, the relatively low flux of 790 photons pulse^−1^ measured by the detector was insufficient to improve the statistical quality of the normalized transmission signal when many shots are averaged. That is to be expected since the *I*_0_ noise-limited statistics are worse than the beamline RMS noise. If we conduct experiments with a limited number of pulses, such as shown in Fig. 5 ▸ with a strongly absorbing calibration foil, the *I*_0_ normalization is beneficial. Improving the efficiency of *I*_0_ detection and reducing readout noise to achieve shot-noise-limited detection with 20% photon absorption would result in less noise than the shot-to-shot fluctuations of the beamline. Due to the low repetition rate of FemtoMAX and the recently improved flux, this would positively influence the accumulation time of the experiments, since the noise of this normalization improves with the flux. One suggested method could be the use of diamond-based transmission diodes, as recently demonstrated at Brookhaven (Keister *et al.*, 2018 ▸).

## 6. Femtosecond XAS experiments at FemtoMAX

**Figure 6:**
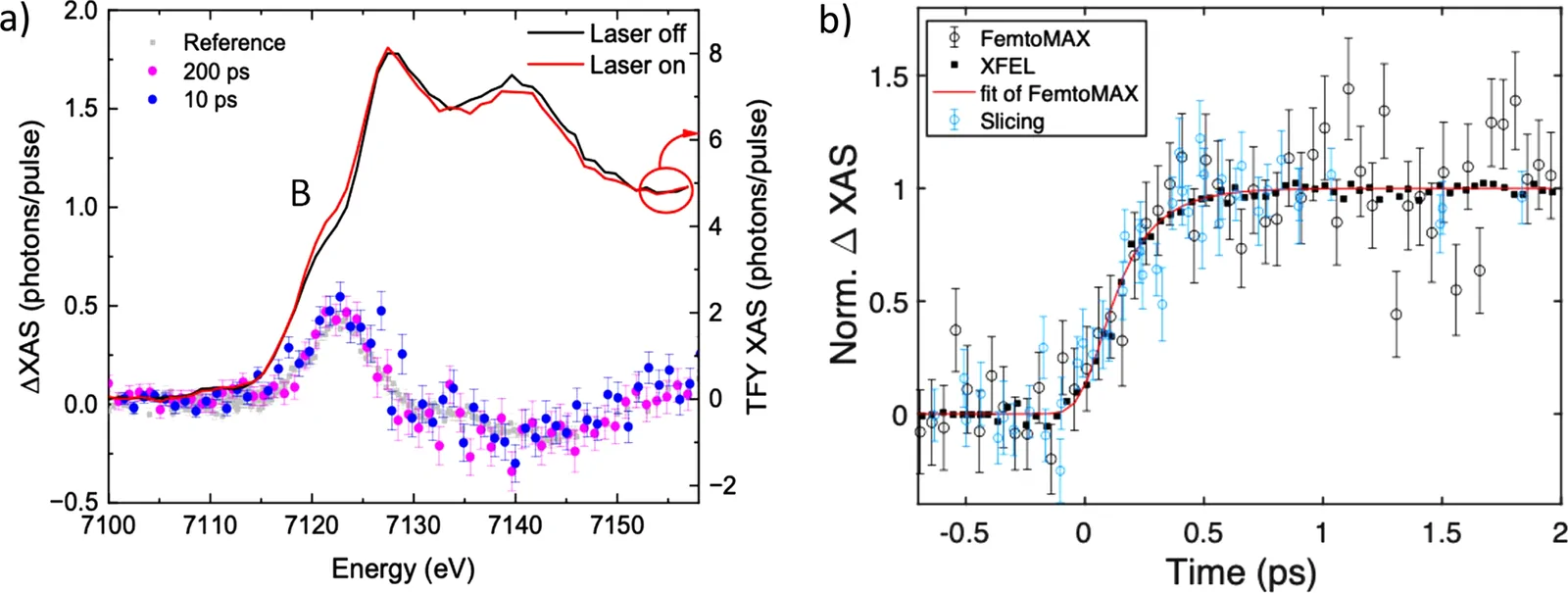
(*a*) Laser on and laser off XAS spectra (solid line) at 200 ps and transient XAS spectra at 10 (blue dots) and 200 ps (magenta dots) along with a reference (gray dots). The left axis shows the normalized absorption; the right axis, to which the red circle and arrow refer, shows the number of fluorescence photons recorded per pulse. The transient signals were multiplied by five and relate to the right axis. (*b*) Time-dependent transient XAS signal recorded in a 1.5 eV window at the B feature, binned to 50 fs (black open circles), compared with data measured at the LCLS (solid black square) and SLS slicing source (blue open circle). The red curve represents a fit of the FemtoMAX data.

We measured X-ray transient absorption spectra of [Fe^II^(bpy)_3_]^2+^ dissolved in water at 50 m*M* concentration in total fluorescence yield mode as a benchmark for the setup. The molecular complex is composed of a central Fe ion coordinated with three bidentate bi­pyridine ligands. This molecule is known to have a low-spin ground state and to relax after optical excitation into the MLCT manifold into its HS quintet state ^5^T within less than 200 fs (Bressler *et al.*, 2009 ▸; Lemke *et al.*, 2013 ▸). This HS state relaxes nonradiatively back into the low-spin ^1^A_1_ ground state within 600 ps at room temperature. Fig. 6 ▸(*a*) shows the measured X-ray absorption spectrum of the ground state and the laser-excited sample after 200 ps. The same figure also compares the transient (fluorescence difference) spectra measured at 10 ps and 200 ps with reference spectra taken at 50 ps and shows an excellent agreement (Gawelda, Pham *et al.*, 2007 ▸; Gawelda, Cannizzo *et al.*, 2007 ▸). The strongest feature is labeled B following the literature. This feature is indicative of the system relaxing into the HS state and should appear with a delay of 1 ps (Bressler *et al.*, 2009 ▸). In our measurements, this feature is slightly broader due to the wider bandwidth of the InSb(111) DCM compared with that of the Si(111) DCM used by Gawelda, Pham *et al.* (2007 ▸).

We recorded a time trace of the transient signal at the B-feature by scanning the laser phase shifter and acquiring data together with both timing tools. Fig. 6 ▸(*b*) compares the measured time trace with data collected during early operation at the XFEL source LCLS in Stanford (Lemke *et al.*, 2013 ▸). The solid red line is the fit of FemtoMAX data using a Gaussian-broadened exponential associate function (Lemke *et al.*, 2013 ▸). The Gaussian corresponds to the IRF, and the exponential term represents the arrival into the HS state. Our analysis results are in line with those from LCLS, considering the significantly lower flux at FemtoMAX. Based on the fit, we find 155 ± 137 fs (LCLS: 163 ± 6 fs, SLS slicing: 200 ± 110 fs) for the exponential time constant and 151 ± 27 fs (LCLS: 155 ± 16 fs, SLS slicing: 250 ± 150 fs) for the Gaussian-shaped IRF. The primary contributor to the timing uncertainty is the S/N. More recently, the sub-30 fs resolution reported by Lemke *et al.* (2017 ▸) was enabled by intrinsically few-tens-of-femtosecond long pulses and shot-to-shot arrival-time correction. Such performance is fundamentally inaccessible at synchrotron-based femtosecond slicing or at FemtoMAX, where the X-ray pulse duration is ∼100 fs and the temporal resolution is dominated by GVM in the liquid jet.

The error bars in the fluorescence difference spectrum [Fig. 6 ▸(*a*)] were calculated based on the fluorescence intensity changes. We used this uncertainty to estimate the X-ray flux shown on the right axis of Fig. 6 ▸(*a*) using the average value and its standard deviation via equation (3) (Saes *et al.*, 2004 ▸; Saes, Gawelda *et al.*, 2003 ▸); we measure an electronic noise and elastic-scattering background level of 0.7 photons pulse^−1^ below the edge.

The statistical distribution of the fluorescence signal in Fig. 6 ▸(*b*) before laser excitation indicates an average of 6.2 (0.08) photons pulse^−1^, while that after time zero was 6.7 (0.08) photons pulse^−1^, including the optical photon leakage of approximately one X-ray photon. Thus, the amount of fluorescence X-rays from the unexcited molecules is 6.2 − 1 − 0.7 = 4.5 (0.1) photons pulse^−1^. The observed difference of 0.5 photons pulse^−1^ corresponds to an excitation fraction of ∼11%. This excitation fraction is in excellent agreement with the values extracted directly from the signal intensity, taking into account the pump power and current laser beam profile.

**Figure 7:**
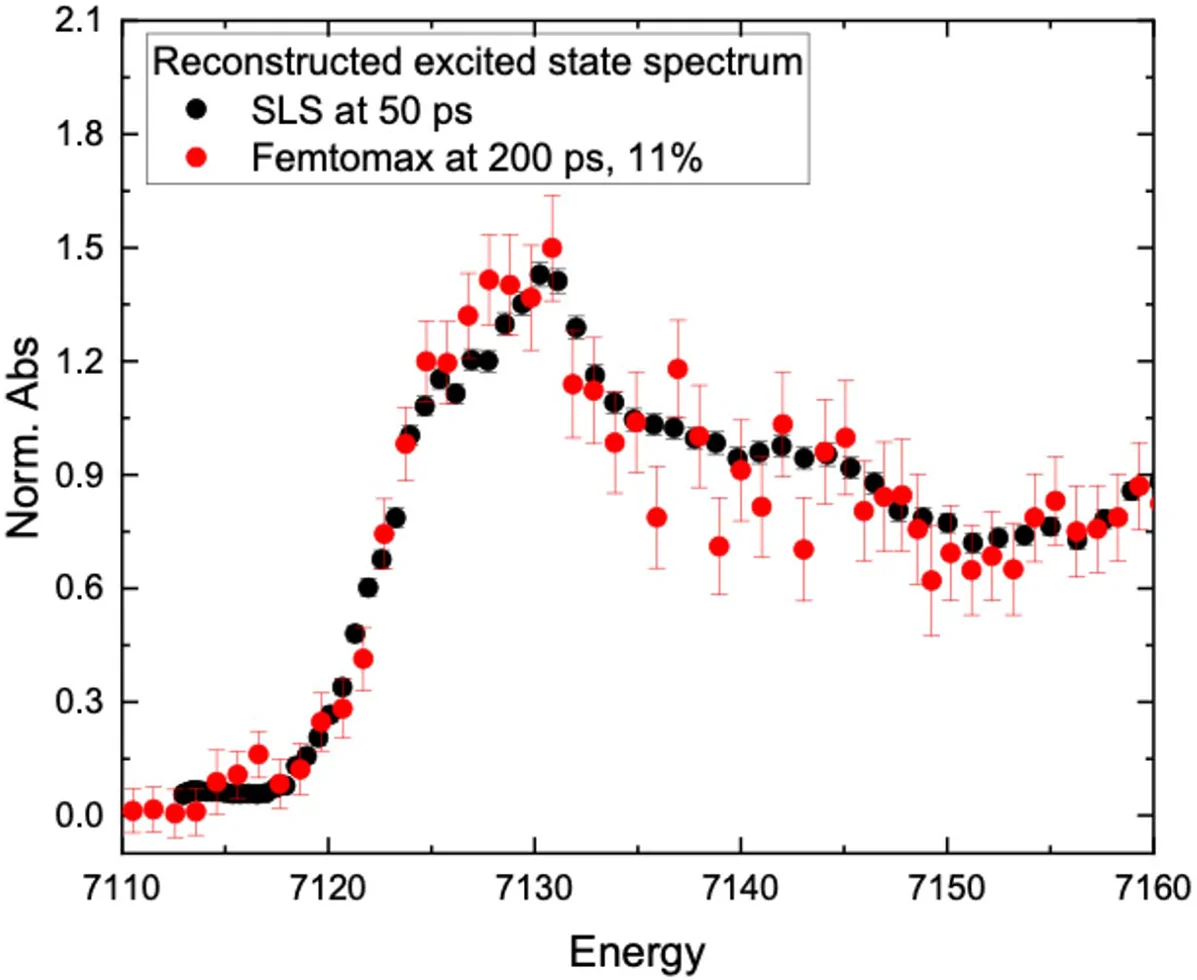
Comparison of the excited-state spectrum at 200 ps measured at FemtoMAX and 50 ps at SLS (Gawelda, Pham *et al.*, 2007 ▸).

Based on this excitation fraction, we can generate the excited-state spectrum from the transient spectrum measured after 200 ps and compare it with literature values (Gawelda, Pham *et al.*, 2007 ▸; Gawelda, Cannizzo *et al.*, 2007 ▸). In this approach, the excited state spectrum is generated via (Bressler *et al.*, 2008 ▸)
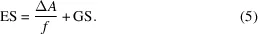
Here, ES and GS represent the spectra of the fully excited (HS) and reactant ground (LS) states, respectively, and Δ*A* is the measured transient absorption spectrum, and *f* denotes the excitation fraction. The resulting spectrum is shown in Fig. 7 ▸, compared with a spectrum measured at the SLS 50 ps after laser excitation. The good agreement within the error margins supports the ability of FemtoMAX to measure detailed structural information of the reacting molecule.

Previous work reported an Fe—N bond length elongation of Δ*R*_Fe–N_ = 0.2 ± 0.02 Å (Gawelda, Pham *et al.*, 2007 ▸; Bressler *et al.*, 2009 ▸) Based on our results we extract within errors the same bond length change of Δ*R*_Fe–N_ = 0.2 ± 0.1 Å.

These results confirm that the XAS endstation delivers an instrument response of 150 fs that is dominated by the GVM in the liquid jet and is capable of resolving ultrafast electronic and structural dynamics of photoactive molecular systems with a reasonable precision, despite operating with a substantially lower flux.

## 7. Comparison of the performance

The time-trace data in Fig. 6 ▸(*b*) were collected using 900 X-ray pulses (90 s accumulation time) per data point. Assuming an incoming X-ray flux of 10^5^ photons pulse^−1^ at 7 keV, this would correspond to 9 × 10^7^ incident photons per data point. Our statistical analysis estimated a slightly lower count of 2.6 × 10^7^ incident photons per data point (see Section 6).

In comparison, the same experiment measured at the SLS slicing femtosecond X-ray source delivered a similar signal-to-noise ratio (∼7) as here at FemtoMAX with a total incident flux of 1.8 × 10^7^ photons per data point. Due to the lower incident photon count per pulse (about 10–12) at a 1 kHz repetition rate (Bressler *et al.*, 2009 ▸), the total collection time per data point for the slicing data was 27 times longer (>40 min) than at FemtoMAX (90 s).

Table 3 ▸ compares the available flux and data accumulation times between the slicing source and FemtoMAX under similar experimental conditions. As a comparison, we include an accumulated flux for picosecond XAS measurement of a 25 m*M* sample taken at SLS in the table (Gawelda, Pham *et al.*, 2007 ▸). The accumulated flux is about 1.6 times higher than we recorded at FemtoMAX and achieves a doubled S/N ratio. The five times lower accumulated flux of the slicing source to achieve the same S/N ratio partly originates from the different excitation yields, 11% at FemtoMAX and 20% at the slicing source. The FemtoMAX laser system is capable of producing higher-power excitation fluences that, with a well defined beam mode, would result in similar, if not better, excitation conditions.

Recent improvements to the beamline showed a higher X-ray flux, and a smaller X-ray focus, 60 µm × 80 µm. With these improvements, the signal quality at FemtoMAX can allow scientific investigations of more dilute solvated samples. At the least we expect a similar S/N (assuming merely an identical excitation fraction) for half the data accumulation time. Improving the laser mode would result in significantly higher potential excitation fractions and much-improved signal strength, at the cost of potentially unwanted side reactions. Adapting the laser excitation angle with respect to the X-ray beam could reduce the group-velocity dispersion mismatch, resulting in a significant improvement in temporal resolution to below 50 fs and represents an attractive perspective.

## 8. Conclusions

FemtoMAX demonstrates the capability to measure ultrafast XANES for studying molecular dynamics with ultrashort temporal precision. With acquisition times of 90 s per data point, we achieved a reasonable signal-to-noise ratio at relatively low excitation fractions and low repetition rate of 10 Hz. The corresponding accumulation times for a time or spectral scan of less than 2 h are well achievable and offer electronic and structural information with 150 fs temporal resolution, limited primarily by group-velocity mismatch in the liquid jet. We have presented a detailed flux and noise analysis based on photon statistics and, finally, suggested several reasonable improvements that could enhance this performance without significant design changes.

## Figures and Tables

**Table 1:** X-ray flux estimated at the sample position by different detectors by integrated electron (current) and photon statistics

Detector (method of measurement)	Measured flux (photons pulse^−1^)	Correction factor	Calculated *I*_0_ (photons pulse^−1^)†
Beamline photodiode (integrated electrons)	1.5 × 10^5^	2/3	1 × 10^5^
*I*_0_ APD (integrated electrons)	773 (gain = 25)‡	Equation (4) and 80% transmission	2.5 × 10^4^ (gain = 25)‡
	386 (gain = 50)‡		1.3 × 10^4^ (gain = 50)‡
*I*_0_ APD (photon statistics)	790	Equation (4) and 80% transmission	2.9 × 10^4^
*I*_1_ APD (integrated electrons)	5.4 × 10^4^	60% through sample	9 × 10^4^
*I*_1_ APD (photon statistics)	2.6 × 10^3^	60%through sample	4.3 × 10^3^
*I*_fl_ fluorescence APD (integrated electrons)	5.3	Equation (4)	1.7 × 10^4^
I_fl_ fluorescence APD (photon statistics)	5.7	Equation (4)	1.8 × 10^4^

† *I*_0_ is determined immediately before the sample.

‡ The *I*_0_ APD gain cannot be determined exactly; it can be between 25 and 50.

**Table 2:** Correction factors used in equation (4) to drive values in Table 1 ▸

	Transmission loss	Fluorescence yield, ϕ_Fe_	Fluorescence yield, ϕ_Mn_	Solid angle, Ω	Transmission, *T*_det_, through Al foil	Detector efficiency, ψ_det_
*I*_0_ Mn	0.2	–	0.3	0.38	0.95	1
Sample Fe	0.014	0.34	–	0.08	0.83	1
*I*_1_	0.2	–	–	–	0.98	1

**Table 3:** Comparison of acquired flux during a time scan for the same signal-to-noise as a femtosecond slicing source and FemtoMAX

	Flux (photons per data point)	Time (s)	Incoming flux (photons per shot)	Repetition rate (Hz)	S/N
Slicing	1.8 × 10^7^	2460	7.3	1000	∼7
FemtoMAX	9 × 10^7^	90	10^5^	10	∼7
Picosecond X-ray pulse	1.4 × 10^8^	140	10^3^	1000	15

## References

[bb1] Alonso-Mori, R., Sokaras, D., Zhu, D., Kroll, T., Chollet, M., Feng, Y., Glownia, J. M., Kern, J., Lemke, H. T., Nordlund, D., Robert, A., Sikorski, M., Song, S., Weng, T.-C. & Bergmann, U. (2015). *J. Synchrotron Rad.* **22**, 612–620. 10.1107/S1600577515004488 PMC4416677 25931076

[bb34] Brandt van Driel, T., Kjaer, K. S., Biasin, E., Haldrup, K., Lemke, H. T. & Nielsen, M. M. (2015). *Faraday Discuss.* **177**, 443–465. 10.1039/c4fd00203b 25675434

[bb2] Bressler, C., Abela, R. & Chergui, M. (2008). *Z. Krist.* **223**, 307–321.

[bb3] Bressler, C. & Chergui, M. (2004). *Chem. Rev.* **104**, 1781–1812. 10.1021/cr0206667 15080712

[bb4] Bressler, C., Milne, C. J., Pham, V. T., Elnahhas, A., van der Veen, R. M., Gawelda, W., Johnson, S., Beaud, P., Grolimund, D., Kaiser, M., Borca, C. N., Ingold, G., Abela, R. & Chergui, M. (2009). *Science* **323**, 489–492. 10.1126/science.1165733 19074309

[bb5] Britz, A., Abraham, B., Biasin, E., van Driel, T. B., Gallo, A., Garcia-Esparza, A. T., Glownia, J., Loukianov, A., Nelson, S., Reinhard, M., Sokaras, D. & Alonso-Mori, R. (2020). *Phys. Chem. Chem. Phys.* **22**, 2660–2666. 10.1039/c9cp03483h 31441480

[bb6] Chen, L. X., Zhang, X. & Shelby, M. L. (2014). *Chem. Sci.* **5**, 4136–4152.

[bb15] Drenth, J. (1999). *Principles of Protein X-ray Crystallography.* New York: Springer.

[bb7] Enquist, H., Jurgilaitis, A., Jarnac, A., Bengtsson, Å. U. J., Burza, M., Curbis, F., Disch, C., Ekström, J. C., Harb, M., Isaksson, L., Kotur, M., Kroon, D., Lindau, F., Mansten, E., Nygaard, J., Persson, A. I. H., Pham, V. T., Rissi, M., Thorin, S., Tu, C.-M., Wallén, E., Wang, X., Werin, S. & Larsson, J. (2018). *J. Synchrotron Rad.* **25**, 570–579. 10.1107/S1600577517017660 PMC5829682 29488939

[bb9] Gawelda, W., Bressler, C., Saes, M., Kaiser, M., Tarnovsky, A. N., Grolimund, D., Johnson, S. L., Abela, R. & Chergui, M. (2005). *Phys. Scr.* **T115**, 102.

[bb10] Gawelda, W., Cannizzo, A., Pham, V., El Nahhas, A., Milne, C. J., Van der Veen, R., Bressler, C. & Chergui, M. (2007). *Chimia* **61**, 179.

[bb11] Gawelda, W., Johnson, M., de Groot, F. M. F., Abela, R., Bressler, C. & Chergui, M. (2006). *J. Am. Chem. Soc.* **128**, 5001–5009. 10.1021/ja054932k 16608334

[bb12] Gawelda, W., Pham, V. T., Benfatto, M., Zaushitsyn, Y., Kaiser, M., Grolimund, D., Johnson, S., Abela, R., Hauser, A., Bressler, C. & Chergui, M. (2007). *Phys. Rev. Lett.* **98**, 057401. 10.1103/PhysRevLett.98.057401 17358897

[bb13] Haldrup, K., Vankó, G., Gawelda, W., Galler, A., Doumy, G., March, A. M., Kanter, E. P., Bordage, A., Dohn, A., van Driel, T. B., Kjaer, K. S., Lemke, H. T., Canton, S. E., Uhlig, J., Sundström, V., Young, L., Southworth, S. H., Nielsen, M. M. & Bressler, C. (2012). *J. Phys. Chem. A* **116**, 9878–9887. 10.1021/jp306917x 22970732

[bb14] Huijser, A., Pan, Q., van Duinen, D., Laursen, M. G., El Nahhas, A., Chabera, P., Freitag, L., González, L., Kong, Q., Zhang, X., Haldrup, K., Browne, W. R., Smolentsev, G. & Uhlig, J. (2018). *J. Phys. Chem. A* **122**, 6396–6406. 10.1021/acs.jpca.8b00916 30052048

[bb16] Katayama, T., Northey, T., Gawelda, W., Milne, C. J., Vankó, G., Lima, F. A., Bohinc, R., Németh, Z., Nozawa, S., Sato, T., Khakhulin, D., Szlachetko, J., Togashi, T., Owada, S., Adachi, S., Bressler, C., Yabashi, M. & Penfold, T. J. (2019). *Nat. Commun.* **10**, 3606. 10.1038/s41467-019-11499-w PMC6689108 31399565

[bb17] Keister, J. W., Cibik, L., Schreiber, S. & Krumrey, M. (2018). *J. Synchrotron Rad.* **25**, 407–412. 10.1107/S1600577517017659 29488919

[bb18] Kroon, D., Nilsson, E., Ahn, B., Bertelli, M., Calarco, R., Clementsson, I., De Simone, S., Ekström, J. C., Jurgilaitis, A., Longo, M., Pham, V.-T. & Larsson, J. (2025). *J. Synchrotron Rad.* **32**, 1052–1058. 10.1107/S1600577525003479 PMC12236248 40455640

[bb19] Kunnus, K., Vacher, M., Harlang, T. C. B., Kjaer, K. S., Haldrup, K., Biasin, E., van Driel, T. B., Pápai, M., Chabera, P., Liu, Y., Tatsuno, H., Timm, C., Källman, E., Delcey, M., Hartsock, R. W., Reinhard, M. E., Koroidov, S., Laursen, M. G., Hansen, F. B., Vester, P., Christensen, M., Sandberg, L., Németh, Z., Szemes, D. S., Bajnóczi, É., Alonso-Mori, R., Glownia, J. M., Nelson, S., Sikorski, M., Sokaras, D., Lemke, H. T., Canton, S. E., Møller, K. B., Nielsen, M. M., Vankó, G., Wärnmark, K., Sundström, V., Persson, P., Lundberg, M., Uhlig, J. & Gaffney, K. J. (2020). *Nat. Commun.* **11**, 634. 10.1038/s41467-020-14468-w PMC6994595 32005815

[bb20] Lemke, H. T., Bressler, C., Chen, L. X., Fritz, D. M., Gaffney, K. J., Galler, A., Gawelda, W., Haldrup, K., Hartsock, R. W., Ihee, H., Kim, J., Kim, K. H., Lee, J. H., Nielsen, M. M., Stickrath, A. B., Zhang, W., Zhu, D. & Cammarata, M. (2013). *J. Phys. Chem. A* **117**, 735–740. 10.1021/jp312559h 23281652

[bb21] Lemke, H. T., Kjaer, K. S., Hartsock, R., van Driel, T. B., Chollet, M., Glownia, J. M., Song, S., Zhu, D., Pace, E., Matar, S. F., Nielsen, M. M., Benfatto, M., Gaffney, K. J., Collet, E. & Cammarata, M. (2017). *Nat. Commun.* **8**, 15342. 10.1038/ncomms15342 PMC5458100 28537270

[bb22] Lima, F. A., Milne, C. J., Amarasinghe, D. C. V., Rittmann-Frank, M. H., van der Veen, R. M., Reinhard, M., Pham, V., Karlsson, S., Johnson, S. L., Grolimund, D., Borca, C., Huthwelker, T., Janousch, M., van Mourik, F., Abela, R. & Chergui, M. (2011). *Rev. Sci. Instrum.* **82**, 063111. 10.1063/1.3600616 21721678

[bb23] Pennacchio, F., Vanacore, G. M., Mancini, G. F., Oppermann, M., Jayaraman, R., Musumeci, P., Baum, P. & Carbone, F. (2017). *Struct. Dyn.* **4**, 044032. 10.1063/1.4991483 PMC5491388 28713841

[bb24] Ponseca, C. S., Chábera, P., Uhlig, J., Persson, P. & Sundström, V. (2017). *Chem. Rev.* **117**, 10940–11024. 10.1021/acs.chemrev.6b00807 28805062

[bb25] Saes, M., Bressler, C., Abela, R., Grolimund, D., Johnson, S. L., Heimann, P. A. & Chergui, M. (2003). *Phys. Rev. Lett.* **90**, 047403. 10.1103/PhysRevLett.90.047403 12570459

[bb26] Saes, M., Gawelda, W., Kaiser, M., Tarnovsky, A., Bressler, C., Chergui, M., Johnson, S. L., Grolimund, D. & Abela, R. (2003). *Synchrotron Radiat. News* **16**(4), 12–20.

[bb27] Saes, M., van Mourik, F., Gawelda, W., Kaiser, M., Chergui, M., Bressler, C., Grolimund, D., Abela, R., Glover, T. E., Heimann, P. A., Schoenlein, R. W., Johnson, S. L., Lindenberg, A. M. & Falcone, R. W. (2004). *Rev. Sci. Instrum.* **75**, 24–30.

[bb28] Schoenlein, R. W., Chattopadhyay, S., Chong, H. H. W., Glover, T. E., Heimann, P. A., Shank, C. V., Zholents, A. A. & Zolotorev, M. S. (2000). *Science* **287**, 2237–2240. 10.1126/science.287.5461.2237 10731140

[bb29] Stamm, C., Kachel, T., Pontius, N., Mitzner, R., Quast, T., Holldack, K., Khan, S., Lupulescu, C., Aziz, E. F., Wietstruk, M., Dürr, H. A. & Eberhardt, W. (2007). *Nat. Mater.* **6**, 740–743. 10.1038/nmat1985 17721541

[bb30] Thompson, A. C., Attwood, D. T., Gullikson, E. M., Howells, M. R., Kortright, J. B., Robinson, A. L., Underwood, J. H., Kim, K.-J., Kirz, J., Lindau, I., Pianetta, P., Winick, H., Williams, G. P. & Scofield, J. H. (2001). *X-ray Data Booklet*, p.1125. Lawrence Berkeley National Laboratory, Berkeley, CA, USA.

[bb31] Tóth, G., Polónyi, G. & Hebling, J. (2023). *Light Sci. Appl.* **12**, 256. 10.1038/s41377-023-01293-1 PMC10593827 37872176

[bb32] Uhlig, J., Fullagar, W., Ullom, J. N., Doriese, W. B., Fowler, J. W., Swetz, D. S., Gador, N., Canton, S. E., Kinnunen, K., Maasilta, I. J., Reintsema, C. D., Bennett, D. A., Vale, L. R., Hilton, G. C., Irwin, K. D., Schmidt, D. R. & Sundström, V. (2013). *Phys. Rev. Lett.* **110**, 138302. 10.1103/PhysRevLett.110.138302 23581383

[bb33] van Bokhoven, J. A. & Lamberti, C. (2016). *X-ray absorption and X-ray emission spectroscopy: theory and applications.* John Wiley & Sons, Inc.

[bb35] Vester, P., Kubicek, K., Alonso-Mori, R., Assefa, T., Biasin, E., Christensen, M., Dohn, A. O., van Driel, T. B., Galler, A., Gawelda, W., Harlang, T. C. B., Henriksen, N. E., Kjaer, K. S., Kuhlman, T. S., Németh, Z., Nurekeyev, Z., Pápai, M., Rittman, J., Vankó, G., Yavas, H., Zederkof, D. B., Bergmann, U., Nielsen, M. M., Møller, K. B., Haldrup, K. & Bressler, C. (2022). *J. Chem. Phys.* **157**, 224201. 10.1063/5.0107224 36546808

[bb36] Zhang, S., Abiven, Y. M., Bisou, J., Renaud, G., Thibaux, G., Ta, F., Minolli, S., Langlois, F., Abbott, M., Cobb, T., Turner, C. J. & Uzun, I. (2017). *Proceedings of the 16th International Conference on Accelerator and Large Experimental Control Systems (ICALEPCS2017)*, Barcelona, Spain, pp. 143–150. TUAPL05.

[bb37] Zhang, X., Wasinger, E. C., Muresan, A. Z., Attenkofer, K., Jennings, G., Lindsey, J. S. & Chen, L. X. (2007). *J. Phys. Chem. A* **111**, 11736–11742. 10.1021/jp0751763 17966996

